# Double Ghost Convolution Attention Mechanism Network: A Framework for Hyperspectral Reconstruction of a Single RGB Image

**DOI:** 10.3390/s21020666

**Published:** 2021-01-19

**Authors:** Wenju Wang, Jiangwei Wang

**Affiliations:** College of Communication and Art Design, University of Shanghai for Science and Technology, Shanghai 200093, China; wangwenju@usst.edu.cn

**Keywords:** double ghost attention mechanism network, double output feature CBAM, optimal nonlocal block

## Abstract

Current research on the reconstruction of hyperspectral images from RGB images using deep learning mainly focuses on learning complex mappings through deeper and wider convolutional neural networks (CNNs). However, the reconstruction accuracy of the hyperspectral image is not high and among other issues the model for generating these images takes up too much storage space. In this study, we propose the double ghost convolution attention mechanism network (DGCAMN) framework for the reconstruction of a single RGB image to improve the accuracy of spectral reconstruction and reduce the storage occupied by the model. The proposed DGCAMN consists of a double ghost residual attention block (DGRAB) module and optimal nonlocal block (ONB). DGRAB module uses GhostNet and PRELU activation functions to reduce the calculation parameters of the data and reduce the storage size of the generative model. At the same time, the proposed double output feature Convolutional Block Attention Module (DOFCBAM) is used to capture the texture details on the feature map to maximize the content of the reconstructed hyperspectral image. In the proposed ONB, the Argmax activation function is used to obtain the region with the most abundant feature information and maximize the most useful feature parameters. This helps to improve the accuracy of spectral reconstruction. These contributions enable the DGCAMN framework to achieve the highest spectral accuracy with minimal storage consumption. The proposed method has been applied to the NTIRE 2020 dataset. Experimental results show that the proposed DGCAMN method outperforms the spectral accuracy reconstructed by advanced deep learning methods and greatly reduces storage consumption.

## 1. Introduction

Hyperspectral imaging is based on numerous narrow-band image data technologies. It combines imaging innovation with spectral technology to enable the detection of the two-dimensional geometric space and spectral information of the target. Hyperspectral imaging uses this type of approach to generate high-resolution continuous narrow-band image data [1]. Hyperspectral images combine the image and spectral information of samples. Image information can reflect external quality characteristics such as the size, shape, and defects of the sample. At a certain wavelength, the image will reflect a certain defect more significantly because different components have different spectral absorptions. The spectral information can fully reflect the differences in the physical structure and chemical composition of the sample, and it has therefore been widely used in face recognition [1], image classification [2], image recognition [3], image restoration [4], and many other applications. However, hyperspectral imaging equipment is expensive, complex, and difficult to move, which limits the further development of hyperspectral imaging research. These problems can be readily solved using RGB to HSI image reconstruction. Hyperspectral imaging technology is therefore a very active area of current research [5].

Spectral reconstruction can be divided into traditional, machine, and deep learning methods. The traditional method is based on statistics such as the pseudo inverse method [6], smoothing inverse method [7], and Wiener method [8]. The accuracy of reconstruction is low and easily affected by noise when the formula transformation is used to reconstruct the spectral matrix.

Therefore, the pseudo inverse method is combined with the adaptive sample selection after due consideration of the method of sample selection [9]. A new transformation is conducted on the verification sample by the adaptive selection of training depending on the spectral similarity of the sample to calculate the reflectance matrix. The natural neighborhood interpolation method [10] is used to reconstruct spectra from different samples, which expands the range of sample selection. However, the local interpolation method [11] estimates the reflectance curve of n-dimensional space from the corresponding tristimulus value, namely, the CIEXYZ or CIELAB sample value, which is also the earliest low-channel to multi-channel spectral study. The weighted coefficient matrix of the locally optimal training sample [12] can also be used to improve the reconstruction accuracy. The performance of spectral reflectance reconstructed from the digital camera is better if the training sample and the test sample are as similar as possible [13]. The method of discrete sine transform (DST) [14] is used to make the spectrum approach the original spectral reflectance and gradually maximizes the approximate value of the spectral original. In this way, it is difficult for the algorithm to set the internal control parameters within a certain range although the accuracy of spectral reflectance can be guaranteed. These traditional methods have simple calculation and are easy to use, but the spectral rebuilding precision is low.

In machine learning, tensor learning is widely used in hyperspectral classification and dimensionality reduction [15,16,17,18]. It has been applied to the latest hyperspectral imaging techniques [19]. Tensor learning uses prior information to calculate the image reconstruction in hyperspectral imaging. There are many other methods of machine learning from low-dimensional to high-dimensional mapping. The primary function [20,21,22] of network training for sample image simulation using sample selection can be used for camera-specific reflection and spectrum mapping between the RGB values and the scene. RGB white balance is used to standardize scene lighting for reflectance-recovery scenarios. However, the use of this method for reconstruction is limited by the sensitivity of the camera sensors. In addition, there are still poor spectral reconstruction results in the case of spectral peaks even in the limited scene. To not be restricted by the camera sensor sensitivity requires the following assumption. A weighted average of the reflectance is selected for all the samples in the training group to remove the restrictions on the camera sensor activity with all the samples and the color of the pixel being smaller than a threshold set for the reconstruction of the spectrum [23]. Recovering high-quality hyperspectral images from RGB based on sparse coding is a fast and low-cost direct method [24,25,26]. Sparse hyperspectral dictionaries are constructed by collecting hyperspectral prior information, which can provide mapping between RGB images and hyperspectral images. However, the accuracy of the reconstruction is affected by the noise of the camera lens, the brightness of the photo, the camera sensor, and the impact of external factors such as the sensor. The sparse reconstruction algorithm is based on the color adaptive dictionary [27]. Three channels of RGB are trained to obtain a three-channel non-negative dictionary using the similarity of spatial content of the single spectral band and RGB images, respectively. The color adaptive dictionary is chosen to improve the sparsity of the dictionary representation by using the color camera in the response amplitudes of the spectral bands. However, some parameter optimization problems affect the spectral reconstruction. Regularization is a basic technique that is robust for solving ill-posed optimization problems and is essential for the reconstruction of hyperspectral images [28]. When the regularization function is combined with the optimization-based network and the complete parameters of the network learnt through end-to-end training, it is possible to overcome the significant calculation problems in the traditional iterative optimization method. However, the details of the image will be lost after spectral reconstruction. Therefore, Akhtar [29] proposed using a space-spectral correlation for hyperspectral training of patch clusters to model the natural spectrum under the Gaussian process. This approach was combined with the RGB image to restore the spectral details from the quantized RGB image of the known spectrum. Although the loss of reconstruction accuracy is relatively reduced, the algorithm network is complicated and this leads to a large amount of calculation.

Deep learning methods are used in hyper spectral image classification and analysis Ref. [30,31,32]. Currently, deep learning has been applied to the reconstruction of RGB hyperspectral images. There are many methods for spectral reconstruction based on deep learning, which mainly use supervised and unsupervised methods. One such method that is unsupervised is generative adversarial networks (GAN) [33,34]. This method requires that the model can effectively capture the structure of data types and consider the spatial context information in RGB images to obtain the spectral reconstruction process. However, in the attempt to construct the spectral data with prior information, the single-pixel-based method cannot effectively utilize the local context when applied to the spectral data. Therefore the reconstructed spectral accuracy is low, the speed is slow, and the running cost is high. Other supervised methods [35] exist such as the convolution neural network (CNN) [36]. In this method, the two-dimensional CNN model mainly focuses on extracting spectral data by only considering spatial correlation. The three-dimensional CNN model uses the relationship between channels to refine the extraction of spectral data. However, the 3D-CNN spectrum reconstruction takes much more time. In addition, there are other supervised networks, such as the scale attention Pyramid (SAPUNet) [37], which uses U-net with an extended convolution [38] to extract features. The accuracy of hyperspectral reconstruction is improved, but the accuracy of spectral reconstruction is only higher in outdoor RGB images, while it is lower in indoor RGB images. The other method is to add a special convolution layer reconstruction network (the 2D convolution batchnorm-relu) on U-NET [39], which can effectively reconstruct hyperspectral images from RGB images. 

In the supervised mode, spectral reconstruction can also be conducted by combining the CNN and 7 × 7 CONv layers [40]. This combination forms a residual structure in the whole network framework. CNN is the core network. The 7 × 7 CONv layer can be regarded as the residual network through which the information of the whole framework is connected. In addition, there are also many different CNNs. The deep residual network HSCNN [41] replaces the remaining blocks with dense blocks, thus forming a new network HSCNN-D. The model greatly deepens the network structure and obtains a more accurate reconstruction. In a multiscale deep CNN proposed by YAN [42] through the symmetric cascade down sampling and up sampling of the intermediate feature map, the local and nonlocal image information can be jointly encoded for spectral representation, and the accuracy of spectral reconstruction can be improved. The network that deepens the convolutional layer is the hierarchical regression network (HRN) [43]. Therefore, residual dense blocks are used to remove image artifacts and global blocks at each level to expand the visual perception of the image. However, these approaches use several kinds of convolution calculations for complex networks and are therefore time consuming. In recent years, popular deep learning frameworks that use attention mechanisms have also been applied to the reconstruction of single RGB images from hyperspectral images. For example, the residual pixel attention network (RPAN) [44], a pixel attention block PA module, is applied to every pixel of all feature maps and adaptively rescales each pixel weight for all input feature maps. Each channel has its own characteristics between channels due to learning interdependence. It is also possible to learn that different positions of a channel have different degrees of characteristics. An adaptive weighted attention network (AWAN) [45] uses a single convolution extracting shallow layer from RGB input features. Next, superimposed multiple double-surplus note blocks, called dual residual attention blocks (DRABs) form a network of deep in-depth feature extractions, through integration of the channel correlation between the characteristic responses of the distribution channel once again. However, the accuracy of the reconstructed hyperspectral images needs to be further improved and the complex calculations for the trained model also occupy a large amount of storage. This is a common problem that is also found in the current RGB reconstructions of HSI images.

We made the following contributions to solve these common problems of low accuracy and excessive consumption of storage:

(1) The framework of the Double Ghost Convolution Attention Mechanism Network (DGCAMN) is proposed. It includes the Double Ghost Residual Attention Block (DGRAB) module, the Double Output Feature CBAM (DOFCBAM), and the optimal non-local area block (ONB). Its purpose is not only single image reconstruction of the hyperspectral image. It must also have the highest precision minimum storage requirements for the operation parameters.
(2) The DGCAMN proposes a Double Ghost Residual Attention Module (DGRAM) that uses GhostNet and PRELU activation functions. It therefore has a lightweight network to reduce the total number of parameters, computational complexity, and storage usage.
(3) The DGCAMN proposes a Double Output Feature CBAM (DOFCBAM), which generates four cross-linked feature vectors in the shared layer of the channel attention mechanism. This maximizes the capture of texture information on the feature graph, which makes the reconstructed hyperspectral image content more abundant.
(4) The DGCAMN proposes the optimal non-local area block. The region with the most abundant feature information in the feature graph could be obtained using the Argmax activation function through reverse evaluation. This not only extracts the structure clues for a long distance, but also maximizes the most useful feature parameters to better improve the accuracy of spectral reconstruction.
(5) In the NTIRE 2020 dataset, the hyperspectral images for single RGB were reconstructed with the most advanced reconstruction accuracy. The storage occupied was the lowest for this approach.

## 2. Our Methods

The double ghost convolution attention mechanism network (DGCAMN) framework workflow is as follows: The RGB image extracts the feature information of the shallow image through a 3 × 3 size convolution kernel. The deeper feature information is extracted using m (m = 8) double ghost residual attention blocks based on the Double Ghost and Double Output Feature CBAM (DOFCBAM) in series superposition. The use of double Ghost can reduce the parameter and computer storage footprint and DOFCBAM is used to reconstruct the accuracy of hyperspectral images. After a 3 × 3 convolution network, the output eigenvalues are added to the original eigenvalues, and the deeper feature information can be output and the shallow information of the original image can be obtained at the same time. The PRELU activation function speeds up the rate of network learning. A 3 × 3 convolution output is performed to ensure the input and output characteristics are the same size. The optimal nonlocal module is entered to enhance the feature connection of the upper and lower layers and to finally reconstruct the high-precision hyperspectral images.

### 2.1. Double Ghost Residual Attention Block (DGRAB)

**Figure 1:**
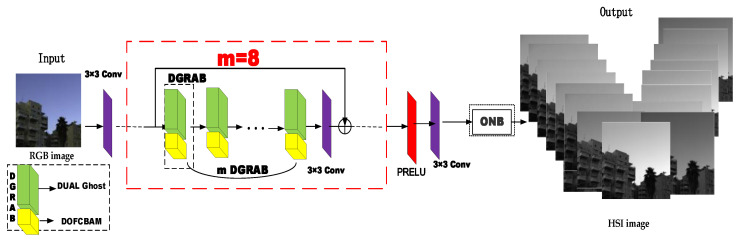
Double Ghost Convolution Attention Mechanism Network framework.

The proposed double ghost residual attention block mechanism is the backbone part of the whole network. It is used in the framework of this article to deepen the role of the network in obtaining deeper spectral characteristic information. The working principle of the DGRAB module shown in Figure 1 is as follows: DGRAB module consists of two ghost residual modules. The first ghost residual block Rm-1 is to deepen the network and extract the feature information of the image at a deeper level.

**Figure 2:**
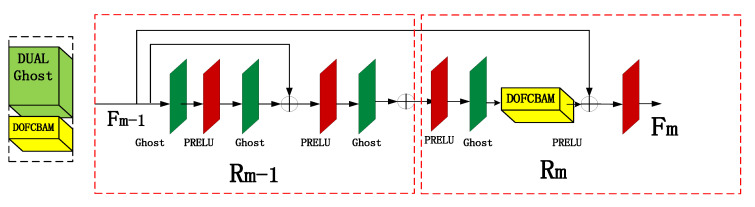
Double Ghost Residual Attention Module.

The purpose of the second ghost residual block Rm is to link the upper characteristic information and to strengthen the link between the global net. The working process of the first residual block in Figure 2 is as follows: First, the features of Fm-1 for the shallow feature information of the image are obtained by 3 × 3 convolution processing. Next, the PRELU activation function through a ghost is conducted with another ghost of the features with the original *F*_*m*−1_ additive. This is effective to capture the characteristics of the original information. After a pair of g host and PRELU activation functions, we get *F*_*m*−1_, which is the formation of the first double ghost residual network *R*_*m*−1_. The second residual block work process is as follows: *F*_*m*−1_ is obtained through the PRELU activation function and ghost to ensure that the output and the original *F_m_* feature input are keep consistent. Next, the result obtained by a convolutional attention mechanism block, the double output feature CBAM, is added to the original feature value. The feature value of *F_m_* is the output of the PRELU activation function. From the second ghost residual module Rm, the process *F_m_* can be expressed as follows:

(1) $$F_{m}=F_{m-1}+\alpha F_{m}(m\in 1,\;\ldots ,\;N)$$

where *α* alpha is the PRELU activation function and *F_m_* is the *m*-th feature image. 

#### 2.1.1. Ghost Network

**Figure 3:**
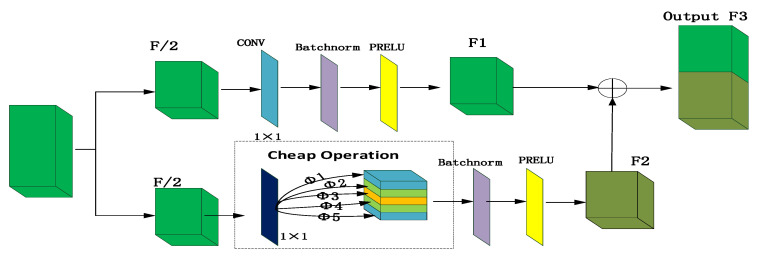
Ghost Network.

The ghost network is introduced into DGRAMN in this article. The total number of parameters and computational complexity required in the ghost network is reduced compared with an ordinary CNN without changing the size of the output feature map [46]. The use of GhostNet solves the problem of large computation and large storage in spectral reconstruction. The ghost network is shown in Figure 3.

In the proposed framework, the ghost network work process is divided into two stages. In the first stage, we propose to averagely divide the original features into two parts. The first part is selected for convolution operation, as seen in Figure 3. First, the feature size is obtained through the convolution operation where the convolution size is 1 × 1. Subsequently, the batch normalization operation is conducted to reduce its value to (0, 1). Next, the PRELU activation function is used to obtain half of the feature graph *F*1. The function of the operation at this stage is to accelerate the convergence of feature learning and avoid the phenomenon of overfitting. The process can be expressed as the following formula:

(2) $$F1=f\times \frac{F}{2}+b$$

where × is the convolution operation and *b* is the bias.

The second stage is to use the cheap operation. We use the depth-wise convolution layer to extract feature information from the second part of the feature map. We set the convolution kernel size of this layer to 1 × 1 to simplify the calculation and do not use bias in the ordinary convolution operation (Equation (3)). A linear operation [46] is used to generate multiple feature images. The generated feature graph is then normalized, which not only retains the original learning features but also accelerates the running time of the hardware training data. In addition, this algorithm reduces the absolute difference between the data, which alleviates the problem of overfitting, and replaces the regular mode of dropout. Finally, the output feature graph of the activation function is *F*2, which can be expressed by the following formula:

(3) $$F2=\Phi (f^{\prime }⊗\frac{F}{2})$$

where ⊗ is one characteristic diagram multiplication, Φ is the linear arithmetic operation, *F*2 is the operation feature and *f*′ is a filter.

In this study, we propose to use the PRELU function as the activation function. The PRELU function only requires small amounts of calculation compared with other activation functions such as tanh and sigmoid. Only a simple linear operation is needed to calculate the error gradient through backpropagation. The calculation time is short and the running speed is fast. In addition, the PRELU function is an example of an ‘unsaturated activation function’, which uses this function to solve the problem of a ‘disappeared’ gradient. This ensures the characteristics of the input value that are less than zero in this case have non-zero value outputs. In the ghost network, the final feature map is the feature graph output from the first stage and the feature graph output from the second stage, which are merged by *F*3 as shown in the following formula:

(4) $$\begin{array}{l} F3=F1+F2\\ =f\times \frac{F}{2}+\Phi (f^{\prime }⊗\frac{F}{2}) \end{array}$$

where × is a convolution, ⊗ is one characteristic diagram multiplication, Φ is the linear arithmetic operation, *F*1 is the common convolution feature, *F*2 is the operation feature and *F*3 is the output feature.

#### 2.1.2. Double Output Feature Convolutional Block Attention Module 

**Figure 4:**
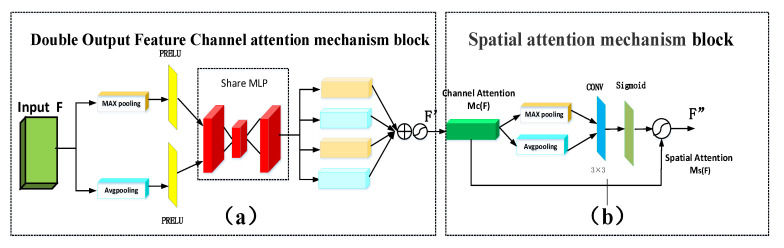
Convolution Attention Mechanism Module Diagram.

A convolutional block attention module (CBAM) is a type of attention mechanism that combines spatial and channel features [47]. It has the ability to focus on the details of the feature target area, which eliminates the role of non-essential information. In the framework of this study, we propose that the double output feature CBAM can effectively extract the feature information of the image, which improves the accuracy of spectral reconstruction. The working principle is shown in Figure 4 and described here. The working process can be expressed by Equations (5) and (6) as follows for a given intermediate feature $F\in R^{C\times H\times W}$:

(5) $$F^{\prime }=M_{c}(F),F\in [H\times W\times C]$$

(6) $$F^{\prime \prime }=M_{s}(F^{\prime })$$

where the input end is the feature graph *F*, *M_c_*(*F*) represents the output feature *F*′ the channel dimension, and *M_s_*(*F*) represents the output feature *F*″ in the spatial dimension.

##### Double Output Feature Channel Attention Mechanism Block

The channel attention mechanism enters the original features $F\in R^{C\times H\times W}$ into the spaces of the global average and maximum pooling layers to give the two channel results (Figure 4a). The purpose of this method is to compress the feature map and to obtain a one-dimensional vector before the operation. Next, the PRELU activation function accelerates the feature learning and reduces the computation time. The shared perception layer share multilayer perceptron (MLP) contains a hidden layer for the size of the vector (r is the reduction ratio). We propose that share MLP computes these two different one-dimensional vectors to generate two output eigenvectors. Therefore, a one-dimensional vector arises from the four-channel attention mechanism $M_{c}\in R^{C\times 1\times 1}$. The first and third double output eigenvectors of the output are $F_{avg}^{c}\in R^{\frac{c}{r\times c}}$. The generation of a double output feature vector can maximize the capture of texture information on the feature map, which makes the reconstructed hyperspectral image more abundant compared with the generation of a feature vector. The second and fourth double output eigenvectors are $F_{max}^{c^{\frac{c\times c}{r}}}$. The generated double output figure feature vector function has the most background information compared with the generation of a feature vector, and therefore reconstructs the hyperspectral image background information more clearly. The four feature vector values are added and then normalized by the sigmoid activation function. The new features after scaling can be obtained using $M_{c}\left(F\right)\in R^{H\times W\times 1}$. This helps to generate the subsequent input characteristics of the spatial attention mechanism module and its working process is represented as follows:

(7) $$\begin{array}{l} F^{\prime }=\sigma (MLP(2\varepsilon Avgpool(F))+MLP(2\varepsilon Maxpool(F)))\\ =M_{c}(F) \end{array}$$

where σ is the sigmoid function and *ε* represents the learning rate of PRELU activation function. MLP is shared by the input of the average pooling and maximum pooling. The avgpool and the maxpool refer to adaptive average pooling and adaptive maximum pooling, respectively.

##### Spatial Attention Mechanism Module

The spatial attention mechanism needs to generate the two-dimensional spatial attention diagram. The network at the spatial level can recognize the feature information of the higher response in the feature graph. (Figure 4b). The first input is the size of the feature $M_{c}\left(F\right)\in R^{H\times W\times 1}$ that goes through the channel attention module. This passes into a one-dimensional channel average pooling layer and maximum pool layer to yield two channel features of the two-dimensional vector $F_{avg}^{s}\in R^{1\times H\times W}$ and $F_{max}^{s^{1\times H\times W}}$, respectively.

This is done according to the channel dimension characteristics by parallel connection two-dimensional vector, which is then passed through a size 3 × 3 convolution kernel f. In our framework, the 3 × 3 convolution is used to reduce the number of parameters without increasing the amount of computation. A sigmoid activation function is used to ensure that the output range of the eigenvalue is between (0, 1), and it is used as the weight coefficient Ms. Finally, the weight coefficient is multiplied by the output features of the original channel attention mechanism $F^{\prime }$. New features are resized according to $M_{s}\left(F\right)\in R^{H\times W\times C}$. This ensures that the finally obtained feature map F is consistent with the original input in the spatial dimension, and its formula is expressed as follows:

(8) $$\begin{array}{l} F^{\prime \prime }=M_{s}(F)\\ =\sigma (f^{3\times 3}\times (Avgpool(F);Maxpool(F)))\\ =\sigma (f^{3\times 3}((F_{avg}^{s},F_{\max}^{s}))) \end{array}$$

where *σ* is the sigmoid operation and $f^{3\times 3}$ represents the 3 × 3 convolution kernel. Here $Avgpool\left(F^{\prime }\right)$ and $Maxpool\left(F^{\prime }\right)$ specifically refer to the output of the adaptive average pooling ($F_{avg}^{s}$) and the adaptive maximum pooling ($F_{max}^{s}$) in the spatial attention mechanism block.

### 2.2. Optimal Nonlocal Block

The nonlocal block (NB) [48,49] has been applied to target detection, segmentation, and other fields. RGB is used to reconstruct the hyperspectral images in the study reported here. The purpose of the non-local block is to enhance the relationship between features. It can directly calculate the relationship between any two positions and it can capture well the spectral feature information. However, the application of NB to the RGB image spectrum reconstruction of HSI generates many parameters, which makes it difficult to obtain the abundant characteristic information accurately. Therefore, we propose an optimal nonlocal block (ONB). If the ONB is compared with ordinary nonlocal plots, it not only extracts clues to long structure but also maximizes access to the most useful characteristic parameters. This helps to improve the accuracy of spectral reconstruction.

**Figure 5:**
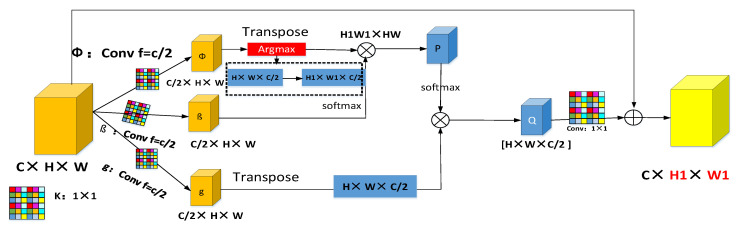
Optimal Nonlocal Block.

The ONB module working process can be divided into two stages. The first stage is as follows: The original feature maps $F_{n,j}$ (C × H × W) is divided into two branches for 1 × 1 convolution kernels. Branch Φ gets C/2 × HW and Branch β receives C/2 × HW (Figure 5). Next, branch Φ transposes the convolution feature and performs the Argmax function (Figure 5) to obtain the most abundant feature information H1 × W1 × C/2. The eigenvalue obtained by the branch is multiplied by the branch β to obtain H1W1 × HW. This gives H1W1 × WH and the weight feature figure P. The second stage is as follows: The original feature map is again subjected to a 1 × 1 convolution to obtain the feature information of branch g: C/2 × HW. C/2 × HW is then transposed. The first phase output feature map P is normalized in batch by softmax and points are multiplied by the transposed feature HW × C/2 to obtain a new eigenvalue Q: H × W × C/2. Q is then added to the original feature graph by a 1 × 1 convolution. The optimal nonlocal blocks can accept any feature size for input. The output therefore retains the same size and the input features. The working process of ONB is as follows:

(9) $$\begin{array}{l} y_{n,j}=\frac{1}{C(F)}\sum_{\forall j}f(F_{n,j},F(\arg\max(F_{n,j}))g(F_{n,j})\\ =\frac{1}{C(F)}\sum_{\forall j}f(F_{n,j},F(\sum_{i}\frac{e^{x_{i}}}{\sum_{j}e^{x_{j}}}i(F_{n,j})))g(F_{n,j}) \end{array}$$

where *n* is the output characteristics of the figure of one position, and *j* represents the index of all possible corresponding positions, that is, *n* gets a nonlocal response value by weighting. *F* represents the input feature graph and $y_{n,j}$ represents the output feature graph. The size of the output feature graph is the same as that of the input feature graph. The f function is used to calculate the similarity of *n* and *j* by calculating the correlation of the *n*th position and all other positions. $g(F_{n,j})$ is used to calculate the representation of the feature map at position *J* for the purpose of information transformation. *C*(*F*) is a normalization function to ensure that the overall information remains unchanged before and after the transformation. $\sum_{\forall j}$ represents a recursive convolution layer with multiple dense connections. $\sum_{i}\frac{e^{x_{i}}}{\sum_{j}e^{x_{i}}}i$ is an Argmax activation function that takes a direct connection from each layer to all subsequent layers.

## 3. Experimental Results and Analysis

### 3.1. Experimental Setup and Evaluation Index

The development environment of this experiment consists of Windows 10, PyTorch version 1.5.1, and two NVIDIA 2080Ti GPUs. The dataset used is the data provided by NTIRE 2020 at https://competitions.codalab.org/competitions/22225. NTIRE 2020 contains the following: 450 training data for 31 channels (400 to 700 nm, one channel per 10 nm) of 512 × 482 and 450 corresponding RGB images; 10 RGB images for a 512 × 482 validation dataset and a hyperspectral image for 31 channels (400 to 700 nm, one channel per 10 nm) of the same size; 10 RGB images for 512 × 482 test datasets. 

The number of double ghost attention modules, m, is 8 and the output channel is 200 in the framework model we designed. The batch size was set as 20, the optimization parameters β1 and β2 were 0.9 and 0.99, respectively, ς was 10^−8^, the DOFCBAM reduction was 16, the ONB reduction was 8, the learning rate was initialized as 0.0001, and the attenuation strategy of the polynomial function as a power function was equal to 1.5. In the process of comparing the proposed method with the YAN [43], HRN [43] and AWAN [45] algorithms, all the experiments were carried out on the same hardware, programming environment and dataset.

**Figure 6:**
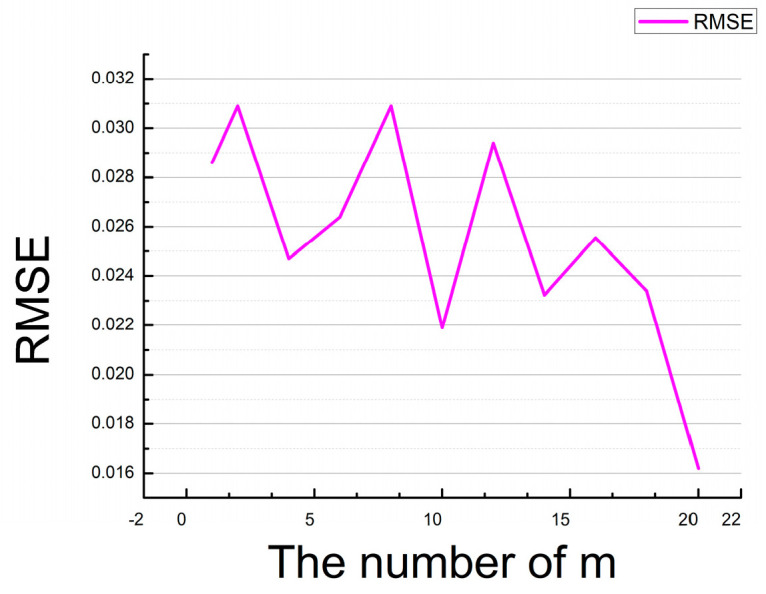
RMSE variation curve with m.

There are two main parameters of this network, m and batch size which will affect the reconstruction HSI from RGB image. m is the double ghost residual attention block (DGRAB). When m = 8, the RMSE value is the smallest. When m ≠ 8, the RMSE value is not the best (see the Figure 6. RMSE variation curve with m). 

**Figure 7:**
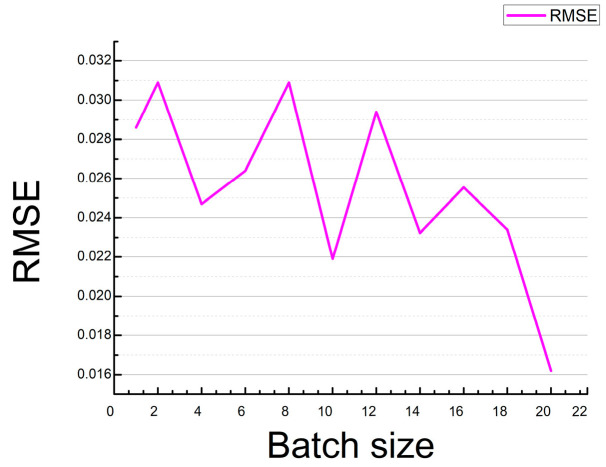
RMSE variation curve with batch size.

Similarly, when batch size = 20, the RMSE value is the smallest in the Figure 7. RMSE variation curve with batch size. The RMSE value is the HSI value of the reconstructed 31 channels, compared with the real HSI value of the 31 channels.

The standard spectral reconstruction indicators used in this study are root mean square error (RMSE) [45], and mean relative absolute error (MRAE) (Equation (11). In Equation (10), the RMSE is the square root of the deviation between the predicted value and the true value and the ratio of the number of observations N. $I_{HSI}^{\left(P\right)}$ refers to the deviation between the reconstructed p_th_ channel value $I_{HSI}^{\left(p\right)}$ and the true spectral value of the p_th_ channel $I_{SR}^{\left(p\right)}$, and is sensitive to outliers in the data. 

In Equation (11), the mean relative absolute error (MRAE) is used to calculate the pixel level parallax between the real image and the reconstructed image, where N represents the whole pixel of the spectral image, and the reconstructed hyperspectral image value $I_{HSI}^{\left(p\right)}$ and the real ground truth $I_{SR}^{\left(p\right)}$. The visual pixel level difference between the two spectral values precisely expressed the build quality of the network. The smaller the values of the two indicators, the better:

(10) $$RMSE=\sqrt{\frac{1}{N}\sum_{p=1}^{N}{\left(I_{HSI}^{\left(P\right)}-I_{SR}^{\left(P\right)}\right)}^{2}}$$

(11) $$MRAE=\frac{1}{N}\sum_{p=1}^{N}\left(\left|I_{HSI}^{\left(P\right)}-I_{SR}^{\left(P\right)}\right|/I_{HSI}^{\left(P\right)}\right)$$

### 3.2. Experimental Analysis

#### 3.2.1. Overall Reconstruction Result Comparison and Evaluation

**Figure 8:**
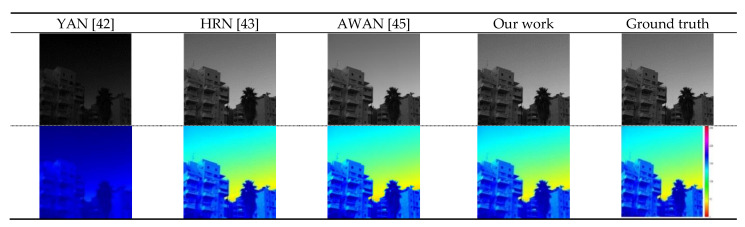
NTIRE 2020 HS verification set for 451 RGB images as determined by YAN, HRN, AWAN, and our method. The reconstructed and real image visualized in the 16th channel map is shown.

The reconstruction result of our algorithm is closer to the real spectral image (Figure 8). The experimental results compared with other advanced algorithms, namely, YAN, AWAN, and HRN, are shown in Table 1. Our RMSE value reaches 0.0162 and the MARE value is 0.0439. Both indexes are the smallest among the evaluation indexes for the listed algorithms. The smaller values of the two indexes correlate with a better performance compared with the other algorithms listed. The RMSE value of our algorithm is reduced by 0.3544 compared with the YAN [42] algorithm. The MRAE decreases by 0.7519. The RMSE value of our algorithm is reduced by 14.28% compared with AWAN [45]; MRAE decreases by 8.15%; the RMSE of our algorithm is reduced by 41.94% compared with the HRN [43] algorithm; MRAE is reduced by 29.60%. This further indicates that the model of the proposed algorithm has the highest accuracy for hyperspectral reconstruction and reaches the most advanced level. Because the ghost network used in our framework is robust, it not only removes a large number of redundant feature images, but also maintains the compactness of multiple ghost networks connecting with adjacent features, thus ensuring the accuracy of spectral reconstruction. In addition, we use the DOFCBAM network to link up and down long-distance feature information, and improve the accuracy of information. The channel attention mechanism module is used to obtain important information on the feature map, and spatial attention is used to obtain the most abundant feature information on the feature map, thus improving the efficiency of spectral reconstruction. In addition, the optimal nonlocal module further enhances the connection between different convolution layers, regardless of the distance. Nonlocal blocks can capture useful feature information and improve the accuracy of spectral reconstruction. However, in the process of our algorithm comparison, HRN, AWAN and YAN do not use a lightweight network, and in the network structure, our algorithm solves the problem that the AWAN training model is too large. Compared with HRN and YAN, our algorithm has higher reconstruction accuracy.

#### 3.2.2. Comparison of Storage Consumption and Lightweight

**Figure 9:**
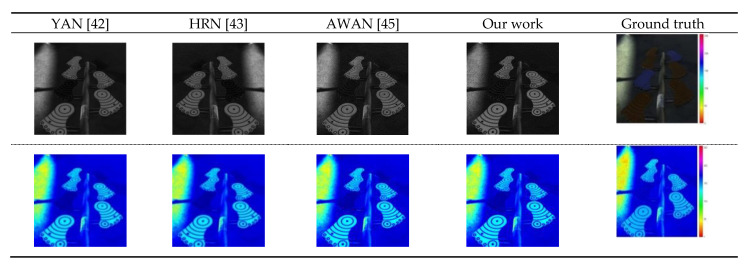
A spectral reversion of the HSI reversion error image in band 31. The analysis uses a validation set for NTIRE 2020.

Our method, YAN, AWAN, and HRN [43] were compared for another NTIRE 2020 hyperspectral validation set for RGB image reconstruction using the same hardware equipment and a batch size set to 20. The visual effect is shown in Figure 9 and the results of the comparison of performances are shown in Table 2.

The outcome of our proposed algorithm reconstruction of the image is closer to the real hyperspectral images (Figure 9). The reason for this better performance is that we employ the ghost network framework, which uses convolution kernels (size 1 × 1). In Table 2’s comparison of our method with the YAN [42], AWAN [45] and HRN [43] algorithms, the model size of the model trained by this method is the smallest, which is 32,898 kb. The proposed DGCAMN framework model greatly reduces the amount of computer hardware storage during RGB to hyperspectral reconstruction and is a lightweight framework model under conditions that produce the same spectral reconstruction accuracy. The YAN, AWAN and HRN, methods occupy 3.17, 6.22 and 3.77 times as much hardware storage as our method, respectively. This situation occurs because the ghost network is used in the proposed DGCAMN framework to replace the traditional convolution in the process of obtaining the features of the image. The simple linear cheap operation is also used to generate more feature information. In the case of the same human visual perception, the size of the ghost network convolution kernel is 1 × 1 in our work compared with other convolution kernels of 3 × 3 and 5 × 5 [46]. This is conducive to the extraction of the local features of the images, but fewer parameters are calculated. The minimum calculation parameters for our algorithm is 2,783,247 KB, which is 1/5, 4/25, and1/38 of the number for the YAN [42], AWAN [45], and the HRN [43] algorithms, respectively (Table 2).

In Table 3, our RMSE value reaches 0.0226 and the MARE value is 0.0750. Both indexes are the smallest among the evaluation indexes for the listed algorithms. The smaller values of the two indexes correlate with a better performance compared with the other algorithms listed. The RMSE value of our algorithm is reduced by 0.042 compared with the YAN [42] algorithm. The MRAE decreases by 0.07. The RMSE value of our algorithm is reduced by 5.39% compared with AWAN [45]; the MRAE decreases by 24.26%; the RMSE of our algorithm is reduced by 37.61% compared with the HRN [43] algorithm; and the MRAE is reduced by 29.20%. This further indicates that the model of the proposed algorithm has the highest accuracy for hyperspectral reconstruction and reaches the most advanced level.

#### 3.2.3. Comparison of Convolution Attention Mechanism Modules

**Figure 10:**
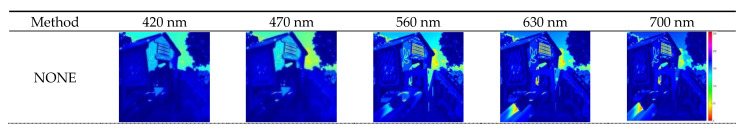

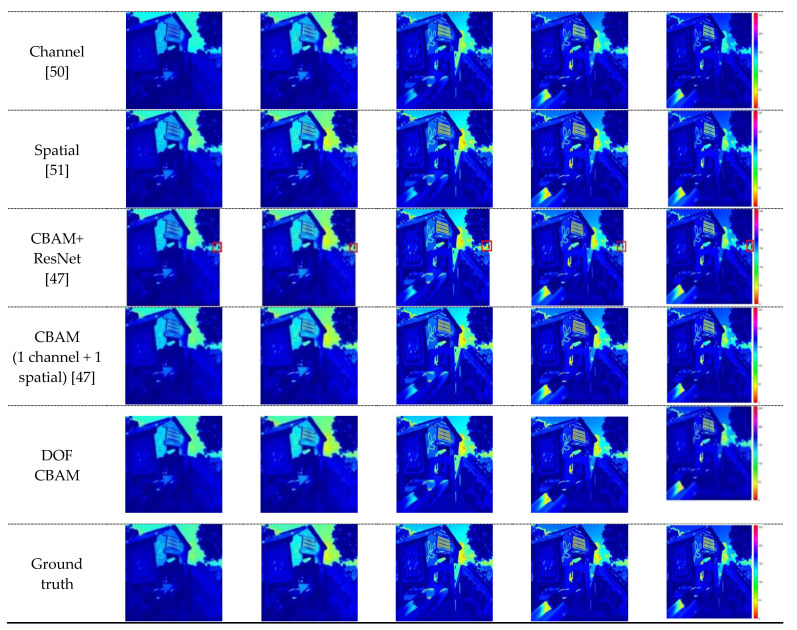
NTIRE 2020 HS validation for NONE, channel, spatial, CBAM, CBAM + ResNet, and DOFCBAM.

The purpose of this experiment is to verify that our double output feature CBAM is superior to other attention modules. The experiments are performed for the same batch size, the same dataset, and the same hardware device. The reconstructed results based on our framework using None, channel attention mechanism [50], spatial attention mechanism [51], the CBAM + ResNet module [47], and Our DOFCBAM at 420, 470, 560, 630, and 700 nm are shown in Figure 10. A comparison of performance is shown in Table 4.

Our DOFCBAM reconstruction of the image effect gives the best fit for the real hyperspectral image compared with the other tested methods (Figure 10). Red artifacts, which are marked by a red box in Figure 10, were evident in the ResNet CBAM [47] analysis for these visualization renderings (Figure 10; CBAM + ResNet [47]). This type of artifact (attention mechanism) was visible from 400 nm to 700 nm and each band of images displayed such problems. The reason for these defects that are highlighted in the small red box is that the center of gravity of the network structure of CBAM + ResNet shifted from the attention mechanism to ResNet during the process of putting CBAM into the ResNet block. In this process, the channel attention mechanism compresses the input feature map into one-dimensional features and loses some feature information. The one-dimensional features are subsequently processed into the mechanism of spatial attention which generates two-dimensional features. Next, the batch normalized operation is performed and combined with the original ResNet network characteristics. However, at this point, the two feature sizes are not the same and cause the CBAM + ResNet spectrum reconstruction with the pseudo-like artifacts shown in Figure 10. Our DOFCBAM has the highest spectral precision (Table 4). The RMSE value is 0.01323 and the MRAE value is 0.07165.

The RMSE value is reduced by 21.25% compared to the outcome when the CBAM algorithm is not used. The MRAE value is reduced by 13.95% compared with the outcome when only the channel attention mechanism channel is used [50]. The RMSE value is reduced by 18.56% and the MRAE value is reduced by 46.28%.

The RMSE values are reduced by 21.71% and the MRAE values are reduced by 17.17% compared with the spatial attentional mechanism channels alone [51]. The RMSE value is decreased by 30.73% and the MRAE value is decreased by 13.99% compared with the CBAM + ResNet algorithm. The RMSE value is reduced by 1.05% and the MRAE value is reduced by 6.71% when a single channel and a mechanism of spatial attention are used compared with our DOFCBAM.

#### 3.2.4. Comparison of Reconstructed Spectral and Analysis

**Figure 11:**
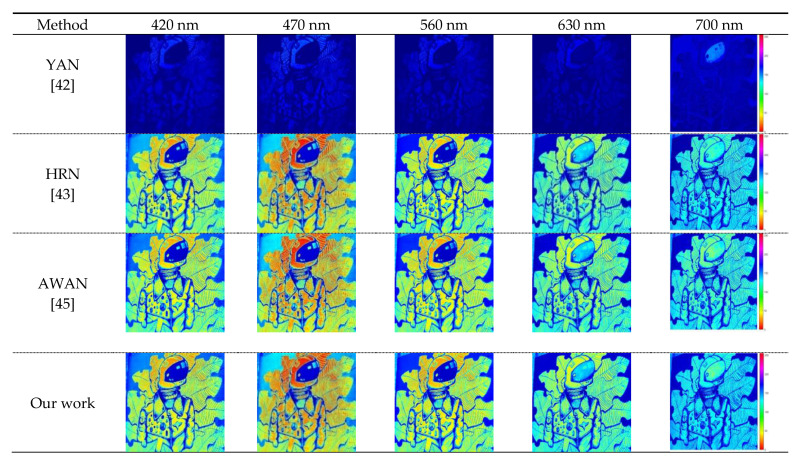

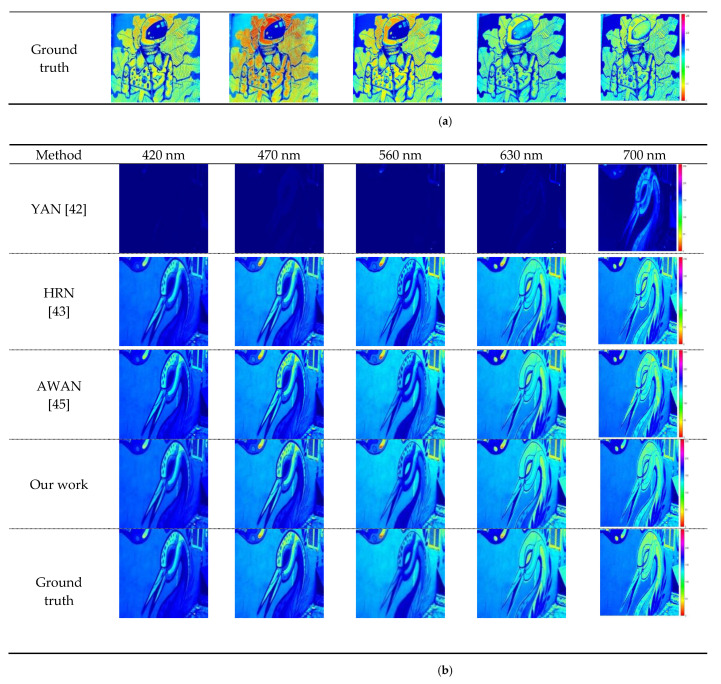
NTIRE 2020 HS validation: (**a**) Visualization for YAN, HRN, AWAN, and our work on the NTIRE 2020 HS validation set. (**b**) Visualization diagrams of YAN, HRN, AWAN, and our work on the NTIRE 2020 HS validation set.

**Figure 12:**
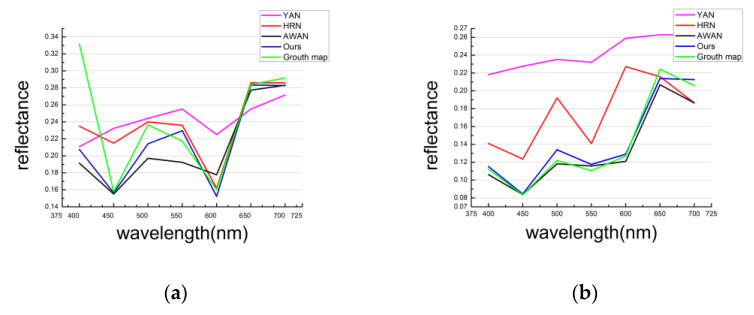
The spectral response curves of multiple spatial points selected from the reconstructed NTIRE 2020 HS verification set. As for Figure 11a: (**a**) Comparison of results as spectral reflectance curves for the validation set of the different algorithms and our work with the NTIRE 2020 HS verification set; As for Figure 11b: (**b**) Comparison of results as spectral reflectance curves for the validation set of the different algorithms and our work with the NTIRE 2020 HS verification set.

The proposed deep-learning model for any of the different RGB images from 400 nm to 700 nm in the high spectrum reconstruction produces images that are very clear and close to the real spectral image (Figure 11a,b). This shows that our algorithm works well. The reflectance curve of our algorithm is blue and the true spectral reflectance curve is green (Figure 12a,b). The reflectance curve of the HRN algorithm [43] is red, the reflectance curve of the YAN algorithm [42] is purple, and the reflectance curve of the AWAN algorithm [45] is black.

Our algorithm has the highest spectral coincidence rate with the true spectral reflectance curve compared with the other three algorithms from 440 nm to 475 nm and from 525 nm to 700 nm (spectral reflectance curve of Figure 12a). The degree of coincidence between the spectral reflectance curve and the real spectral reflectance curve of our algorithm is also the highest compared with the other three algorithms shown in Figure 12b The accuracy of our algorithm for the reconstruction of the image is therefore the highest compared with other algorithms.

The DGRAB series when m is eight can be used to extract in-depth feature information. Our Double Output Feature CBAM can maximize the capture of texture details on the feature maps, which makes the reconstructed hyperspectral images richer and clearer. In addition, our Argmax function (Section 3.2.4) can accurately obtain the useful feature size. The non-local block can effectively connect the information of the convolution layer with the adjacent and different positions outside the adjacent area, thus maintaining more feature information.

Our algorithm framework to rebuild the spectrum characteristics achieves an advanced level of performance and achieves a best fit for the spectrum information curve.

## 4. Conclusions

In this study, we propose the DGCAMN framework for hyperspectral image reconstruction as a way to generate higher-quality images. The DGCAMN framework works by reducing the number of parameter calculations and deploys a large number of storage -training modules to solve the RGB image reconstruction of the hyperspectral image. In future studies, we will try to reduce the noise associated with the reconstruction of the RGB to further improve the DGCAMN framework. At the same time, we will be implementing this approach for mosaic or line hyperspectral cameras, where the deep learning algorithm would fill in the spectral gaps at specific spatial points.

## Figures and Tables

**Table 1:** A quantitative comparison of different algorithms and our method for the NTIRE 2020 hyperspectral verification set. The best results are highlighted in bold.

Method	RMSE	MRAE
YAN [42]	0.3706	0.8009
AWAN [45]	0.0189	0.0478
HRN [43]	0.0279	0.0696
**Our work**	**0.0162**	**0.0439**

**Table 2:** Comparison of different algorithms and our work for analysis of the NTIRE 2020 dataset.

Method	Model Size	Model Size Ratio	Parameter
YAN [42]	104,304 KB	3.17	102 G
AWAN [45]	204,690 KB	6.22	17,461,521 KB
HRN [43]	123,879 KB	3.77	164.01 G + 31.705 M
**Our work**	**32,898 KB**	**1**	**2,783,247 KB**

**Table 3:** Quantitative comparison of different algorithms and our method for the NTIRE 2020 hyperspectral verification set. The best results are highlighted in bold.

Method	RMSE	MRAE
YAN [42]	0.0646	0.1345
HRN [43]	0.0239	0.0969
AWAN [45]	0.0311	0.0932
**Our work**	**0.0226**	**0.0750**

**Table 4:** Comparison of different frame structures and our work on the NTIRE 2020 HS verification set.

Method	RMSE	MRAE
None	0.0168	0.0889
Channel [50]	0.0279	0.1334
Spatial [51]	0.0169	0.0865
CBAM + ResNet [47]	0.0191	0.0834
CBAM [47]	0.01337	0.0763
**Our DOFCBAM**	**0.01323**	**0.07165**

## Data Availability

The dataset used is the data provided by NTIRE 2020 at https://competitions.codalab.org/competitions/22225.
